# Shifts in the Active Rhizobiome Paralleling Low *Meloidogyne chitwoodi* Densities in Fields Under Prolonged Organic Soil Management

**DOI:** 10.3389/fpls.2019.01697

**Published:** 2020-01-10

**Authors:** Paula Harkes, Joris Johannes Matheus van Steenbrugge, Sven Johannes Josephus van den Elsen, Afnan Khalil Ahmad Suleiman, Johannes Jan de Haan, Martijn Hermanus Maria Holterman, Johannes Helder

**Affiliations:** ^1^ Laboratory of Nematology, Department of Plant Sciences, Wageningen University & Research, Wageningen, Netherlands; ^2^ Department of Microbial Ecology, NIOO-KNAW, Wageningen, Netherlands; ^3^ Department of Microbiological Water Quality and Health, KWR Watercycle Research Institute, PE Nieuwegein, Netherlands; ^4^ Open Teelten, Department of Wageningen Plant Research, Wageningen University & Research, Lelystad, Netherlands

**Keywords:** organic soil management, active microbiome, rhizosphere, disease suppressiveness, *Meloidogyne chitwoodi*

## Abstract

Plants manipulate their rhizosphere community in a species and even a plant life stage-dependent manner. In essence plants select, promote and (de)activate directly the local bacterial and fungal community, and indirectly representatives of the next trophic level, protists and nematodes. By doing so, plants enlarge the pool of bioavailable nutrients and maximize local disease suppressiveness within the boundaries set by the nature of the local microbial community. MiSeq sequencing of specific variable regions of the 16S or 18S ribosomal DNA (rDNA) is widely used to map microbial shifts. As current RNA extraction procedures are time-consuming and expensive, the rRNA-based characterization of the active microbial community is taken along less frequently. Recently, we developed a relatively fast and affordable protocol for the simultaneous extraction of rDNA and rRNA from soil. Here, we investigated the long-term impact of three type of soil management, two conventional and an organic regime, on soil biota in fields naturally infested with the Columbian root-knot nematode *Meloidogyne chitwoodi* with pea (*Pisum sativum*) as the main crop. For all soil samples, large differences were observed between resident (rDNA) and active (rRNA) microbial communities. Among the four organismal group under investigation, the bacterial community was most affected by the main crop, and unweighted and weighted UniFrac analyses (explaining respectively 16.4% and 51.3% of the observed variation) pointed at a quantitative rather than a qualitative shift. LEfSe analyses were employed for each of the four organismal groups to taxonomically pinpoint the effects of soil management. Concentrating on the bacterial community in the pea rhizosphere, organic soil management resulted in a remarkable activation of members of the Burkholderiaceae, Enterobacteriaceae, and Pseudomonadaceae. Prolonged organic soil management was also accompanied by significantly higher densities of bacterivorous nematodes, whereas levels of *M. chitwoodi* had dropped drastically. Though present and active in the fields under investigation Orbiliaceae, a family harboring numerous nematophagous fungi, was not associated with the *M. chitwoodi* decline. A closer look revealed that a local accumulation and activation of *Pseudomonas,* a genus that includes a number of nematode-suppressive species, paralleled the lower *M. chitwoodi* densities. This study underlines the relevance of taking along both resident and active fractions of multiple organismal groups while mapping the impact of *e.g.* crops and soil management regimes.

## Introduction

For decades, conventional soil management has resulted in consistent and high level of crop production by external inputs such as chemical fertilizers and pesticides. However, it is widely acknowledged that intensive monocropping has a number of downsides including soil degradation, leaching of nutrients, and biodiversity loss (Tsiafouli et al., 2014). Organic farming, an umbrella term for a wide range of management regimes having the abstinence of the use of mineral fertilizers and chemical pesticides in common, is a possible alternative that might alleviate the negative impact of crop production on soil ecosystems. In organic farming, most often organic manure is used to replenish the nutrient levels in the top soil and to maintain or increase the overall soil organic matter content. In addition, grain legumes are frequently part of the crop rotation because of their nitrogen binding capability. However, especially in Europe a wider application of grain legumes is currently hampered, by the relatively high level of variability in yield. This variation is thought to be due to the sensitivity of these crops to biotic and abiotic stressors (Cernay et al., 2015).

One of the key characteristics of sustainable soil management regimes should be the preservation of a relatively high level of soil biodiversity. In terms of biomass, bacteria and fungi are the most important biotic constituents of soils. Depending on soil type, cultivated soils typically harbor 0.2–0.7 mg of bacteria per g of dry soil, whereas the fungal community is represented by 0.01–0.2 mg per g (Kaczmarek, 1984). Protists and nematodes are major consumers of bacteria and fungi in soil ecosystems. Although the biomass of protists and nematodes is small compared to the primary decomposers (Bar-On et al., 2018), their impact on the turnover of bacteria and fungi is enormous. Protists alone are typically consuming >50% of the bacterial productivity (Foissner, 1999). Though it is a simplification of the biological reality, one could argue that the bacterial and fungal communities are shaped by (1) the quantity and nature of external C and energy inputs into the soil ecosystem, and (2) the activity of protist and nematode communities.

Being present in the soil ecosystem does not imply that a given organism is actively participating in the soil food web. On the contrary, many soil inhabitants are able to reduce their metabolic activity when unfavorable conditions occur, such as food scarcity or drought. This is especially relevant for bulk soils, where typically 80% of the cells, and 50% of the Operational Taxonomic Units (OTUs) are inactive (Lennon and Jones, 2011). Hence, it is essential to take both the resident and the active fractions into account when assessing the biological functioning of a soil ecosystem. A range of studies underlined the relevance of the distinction between resident and active soil biota (Baldrian et al., 2012; Nunes et al., 2018; Schostag et al., 2019). For taxonomic profiling, 16S or 18S ribosomal DNA (rDNA) is often used as molecular marker. Ribosomal RNA is frequently used to map the active microbial fractions (Ofek et al., 2014; De Vrieze et al., 2016). By the molecular characterization of both the resident and the active fractions of the bacterial, fungal, protist, and metazoan community, it is possible to assess the impact of soil management regimes on the soil food web (Harkes et al., 2019).

The rhizosphere of plants creates a center of high metabolic activity in soils. At the interface between the plant root and soil, the plant releases primary and secondary metabolites (Hinsinger et al., 2009; Reinhold-Hurek et al., 2015). With this blend of plant-derived components, the plant boosts a specific fraction of the soil biota. In return, stimulated microbiota increase the bioavailability of plant nutrients and/or they may contribute to the protection of the plants against pathogens (Lugtenberg and Kamilova, 2009; Berendsen et al., 2012; Turner et al., 2013). Especially in agricultural soil, the microbial community structure was shown to be distinct from the surrounding bulk soil (Sharma et al., 2005). Due to the application of fertilizers, root exudation is enhanced which on its turn affects the microbial community in the rhizosphere (Zhu et al., 2016).

Next to bacterivores and fungivores, the nematode community harbors a wide range of plant parasites. Most of them are relatively harmless root hair feeders and ectoparasites (Quist et al., 2015). Only a small subset may have a high impact in crop production. Root-knot nematodes (RKN), members of the genus *Meloidogyne*, are number one in terms of global crop damage by plant-parasitic nematodes (Jones et al., 2013). The highly polyphagous Columbian RKN *Meloidogyne chitwoodi* has a global distribution in temperate climate zones (Santo, 1989). In this study we investigated the long-term effects of three soil management regimes, conventional, integrated and organic, on the soil microbiome in fields naturally infested with *M. chitwoodi.* The legume *Pisum sativum* was used as main crop in these fields. Illumina MiSeq sequencing was used to characterize the active (rRNA) as well as the resident (rDNA) communities of bacteria, fungi, protozoans and metazoans both in bulk soil and in the rhizosphere. The main objectives of this study were (i) to characterize the resident and active microbial community in the rhizosphere of pea with the underlying hypothesis that—besides being present—microbiota need to be active in order to be able to contribute to local changes in food web functioning, (ii) to map the effects of pea on the active and resident fractions of the four organismal group in the rhizosphere compared to the bulk soil under different soil management systems, and (iii) to identify microbial taxa which activities changed in parallel with distinct infestation levels of the root-knot nematode *M. chitwoodi*

## Materials and Methods

### Study Sites

Samples were collected at the WUR experimental farm “Vredepeel”, which is located in the south east of the Netherlands (51°32N and 5°51E). Experimental plots were situated on sandy soils (93,3% sand, 4.5% silt, 2.2% clay) with an organic matter (OM) content of 3%–5%. Three different soil management regimes were continuously applied from 2001 onwards: “ConMin,” “ConSlu” and “Org.” ‘“ConMin fields” solely received mineral fertilizer and processed organic fertilizer (liquid mineral concentrates), whereas “ConSlu fields” were supplemented with mineral fertilizer and slurry (pig/cow). In case of organic soil management (“Org fields”), farmyard manure and cow slurry were applied, and no pesticides were used. For further details of the set up and layout of the soil management experiments see the research reports (De Haan et al., 2018a; De Haan et al., 2018b).

### Soil Sampling

Pea (*P. sativum*) is one of the main crops in the crop rotation system and was sown on the 10^th^ of April 2018. Sampling was executed twice, during the vegetative stage (7^th^ of May) and during the generative stage (31^st^ of May). Each of the three no-tillage fields (ConMin, ConSlu, and Org) was divided in 6 subfields of 540 m^2^ (**Supplementary Figure S1**). In each subfield, a bulk soil and a rhizosphere sample was taken, resulting in six pseudo-replicates. Rhizosphere composite samples were taken by harvesting all pea plants within a square of 20 x 20 cm. A spade of 20 cm was used to carefully lift the soil and take out the all pea plants, this was done in triplicate for each subfield. Excessive soil was removed by shaking the plants and whole plants were transported to the laboratory at the field site. At the field laboratory, the remaining soil that adhered to the roots was brushed off from 10 individual pea plants. Bulk soil was collected by combining three individual cores in the close vicinity of the rhizosphere sampling spot. This was done in between the pea rows with the use of an auger (ø1.5 cm, depth approximately: 15 cm). In total 36 samples (18 rhizosphere and 18 bulk) were taken at each time point, making a total of 72 samples.

Rhizosphere and bulk soil samples were frozen in liquid nitrogen and transported on dry ice to the laboratory, and stored at -80°C until further processing.

For nematode extraction a composite sample was collected in each field (ConMin, ConSlu, and ORG). In total two soil cores per subplot were randomly sampled (ø1.5 cm, depth approximately: 15 cm) resulting in 12 soil cores per field. Composite samples were mixed and stored at 4°C. Two days after collection, nematodes were extracted from 100 g soil, using an elutriator (Oostenbrink, 1960). This was done *in duplo* for each field resulting in six nematode suspensions.

### DNA/RNA Extraction and cDNA Synthesis

Both DNA and RNA were simultaneously extracted from soil samples (2 g each), using a lab-made protocol based on phenol-chloroform-isoamylalcohol extraction (Harkes et al., 2019). Quality and quantity of the obtained RNA and DNA was measured with a Nanodrop and Qubit. The nucleic acid eluate was stored at -80°C until further processing. For synthesis of cDNA from extracted RNA, the Maxima First Strand cDNA Synthesis Kit for RT-qPCR (Fermentas, Thermo Fisher Scientific Inc., USA) was used according to the manufacturer’s instructions. All individual DNA and cDNA samples were diluted to 1 ng/µl and 0.1 ng/µl, respectively, and used as template for PCR amplification.

To estimate the nematode density, a subsample of the nematode suspension (1/10 of each sample) was counted under a dissecting microscope. This was done in triplicate. Hereafter, nematode suspensions were concentrated and lysed according to Vervoort et al. (2012). This resulted in 100 µl purified DNA, which served as a template for quantitative PCR (qPCR).

### PCR Amplification and Sequencing

The variable V4 region of bacterial 16S rRNA gene was utilized as target for the analyses of Illumina 16S rDNA sequencing and the V9, V7–V8, V5–V7 regions were utilized as targets for protozoa, fungi, and metazoan 18S rDNA sequencing, respectively. To prepare the samples for sequencing, a two-step PCR procedure was followed as described in (Harkes et al., 2019). In short, locus-specific primer combinations extended with an Illumina read area and the appropriate adapter were used to produce primary amplicons. This was done in triplicate for all samples and for each of the four organismal groups. PCR 2 was conducted on 40x diluted amplicons of PCR1 to attach the Illumina index and the Illumina sequencing adaptor. Products of PCR 1 and 2 were randomly checked on gel to ensure amplification was successful. Finally, PCR products of fungi, protozoa and Metazoa were pooled and sent for sequencing. Bacterial PCR products were sent separately in order to improve the sequencing resolution. Sequencing was done at Bioscience—Wageningen Research, Wageningen, Netherlands—using the Illumina MiSeq Desktop Sequencer (2*300nt paired-end sequencing) according to the standard protocols.

For analysis of the obtained nematode DNA from the 100g subsamples, 12 nematode taxa were selected for qPCR. 11 primer sets to asses a various set of plant parasitic nematodes—including *M. chitwoodi*—and one primer set to measure the total nematode density (see **Supplementary Table S1**).

### Bioinformatics Framework

The composition of microbial communities of the soil samples was analysed based on the sequencing data obtained from the Illumina MiSeq platform. Reads were sorted into the experimental samples according to the unique combination of two index sequences. Thereafter, reads were sorted into the four organismal groups based on their locus-specific primer sequences.

Forward and reverse reads were paired for bacteria and fungi, while single-end (forward) sequences were analysed for protozoa and metazoan. The four taxonomical groups were quality trimmed by BBDUK and then merged *via* VSEARCH (Rognes et al., 2016; Bushnell, 2018). Resulting unique sequences were then clustered at 97% similarity by using the usearch_global method implemented in VSEARCH and a representative consensus sequence per *de novo* OTU was determined (Rognes et al., 2016). The clustering algorithm also performs chimera filtering to discard likely chimeric OTUs with UCHIME algorithm in *de novo* mode (Edgar et al., 2011) implemented in VSEARCH. Sequences that passed quality filtering were then mapped to a set of representative consensus sequences to generate an OTU abundance table. Representative OTU sequences were assigned to a taxonomic classification *via* BLAST against the Silva database (version 12.8) for bacteria, fungi, and metazoan and PR2 database (Guillou et al., 2013) for protozoa using SINA (Pruesse et al., 2012). Sequences belonging to chloroplasts, cyanobacteria, and mitochondria were discarded from the bacterial dataset; sequences not belonging to Fungi and Metazoa were removed for 18S Fungi and Metazoa datasets, respectively and Streptophyta, Metazoa, fungal, and unclassified Opisthokonta sequences were filtered for Protozoa dataset. Low-abundance OTUs (those with abundance of <0.005% in the total data set) were discarded prior to analysis (Bokulich et al., 2013). Samples were transformed using Hellinger transformation for all downstream analyses.

### Processing and Analysis of Nematode Specific Sequences

For the nematode specific analysis, metazoan reads were blasted against a nematode database after quality trimming of the reads. Trimmomatic v.0.35 (Bolger et al., 2014) was used to trim poor quality bases (four base sliding window with a 13 (p = 0.05) average phred score cut-off), remove the locus specific primer and filter out short reads (<50 bases). The Blast database was based on the dataset of Holterman et al. (2017). Forward and reverse reads were blasted separately as the sequences did not overlap. Each read had one or multiple families assigned to it based on the best blast hit and any additional hits that differed by no more than one base pair from the best blast hit. Reads with less than 92% identity to the sequences in the database were considered not to be nematodes. Reads between 92% and 95% identity were counted as nematodes, but no family name was assigned to them. After this the results for the forward and reverse sequence of each mate pair was compared. Where possible, the results of both reads were combined to refine the family assignment. If the family assignments of both reads of a mate pair were in complete disagreement, the reads were discarded. In some cases, the amplified SSU fragment did not allow for the distinction between certain families, and the reads had to be pooled into a larger taxonomic unit, e.g. all members of the order Dorylaimida or members of the families Bastianiidae and Prismatolaimidae.

### Statistical Analysis

Good’s coverage was assessed (Good, 1953) in order to estimate what percent of the total species is represented in each sample. We explored β diversity patterns by performing principal coordinate analysis (PCoA) with Bray-Curtis dissimilarity using QIIME software (Caporaso et al., 2012). Permutational multivariate analysis of variance (PERMANOVA) was used to compare the microbial community structure between soil managements taken from different sites and with different plant growth stages for active and resident community for four different taxa. This was performed with 1,000 permutations using the adonis function, based on Bray-Curtis distances using the “vegan” package (Oksanen et al., 2015) in R. In order to compare microbial community diversity in a phylogenetic context, UniFrac was performed with 1,000 permutations *via* the “phyloseq” package in R (Mcmurdie and Holmes, 2013). To assess variation in both relative abundance and presence/absence, we analysed both weighted and unweighted UniFrac distances (Lozupone et al., 2007). To investigate the indicator taxa involved in the differences between resident and active community, a linear discriminate analysis (LDA) effect size (LEfSe) was conducted in Microbiome Analyst (Dhariwal et al., 2017) to explore the differential microbial populations at the family level for the four different taxa (Segata et al., 2011). A significance level of α ≤ 0.05 was used for all biomarkers evaluated in this study.

To assess differences between family read abundances of nematodes, a Kruskal–Wallis test was conducted, followed by a Dunn’s Test to test for significance between each of the three management types (IBM SPSS Statistics 25).

## Results

The long-term impact of organic soil management on four major soil organismal groups was monitored in experimental field where pea was grown as main crop. The bacterial and fungal communities were mapped as main primary decomposers, whereas protists and metazoa (mainly nematodes) were included as representatives of the next trophic level. For each of the four organismal groups resident and the active communities were characterized in (1) bulk and rhizosphere soil, (2) with three types of soil management, and (3) at two time points representing the vegetative and the generative growth stage of pea.

### General Analyses of the Sequencing Data

Total DNA and RNA was extracted from 72 bulk soil and rhizosphere samples. MiSeq sequencing was performed on ribosomal DNA and cDNA fragments (16S for bacteria or 18S for fungi, protists, and Metazoa). After filtering, a total of 22 million sequences with an average length of 250bp were retained, comprehending 208 samples for all taxa together. Comprehensive sampling of the microbial community was performed for all treatments, with average sequence coverage of 93%, 95%, 99%, and 99% for protozoa, bacteria, fungi, and metazoan, respectively determined by Good’s coverage estimate.

### Difference Between Resident and Active Communities In Bulk and Rhizosphere Soil at Organismal Group Level

**Figure 1:**
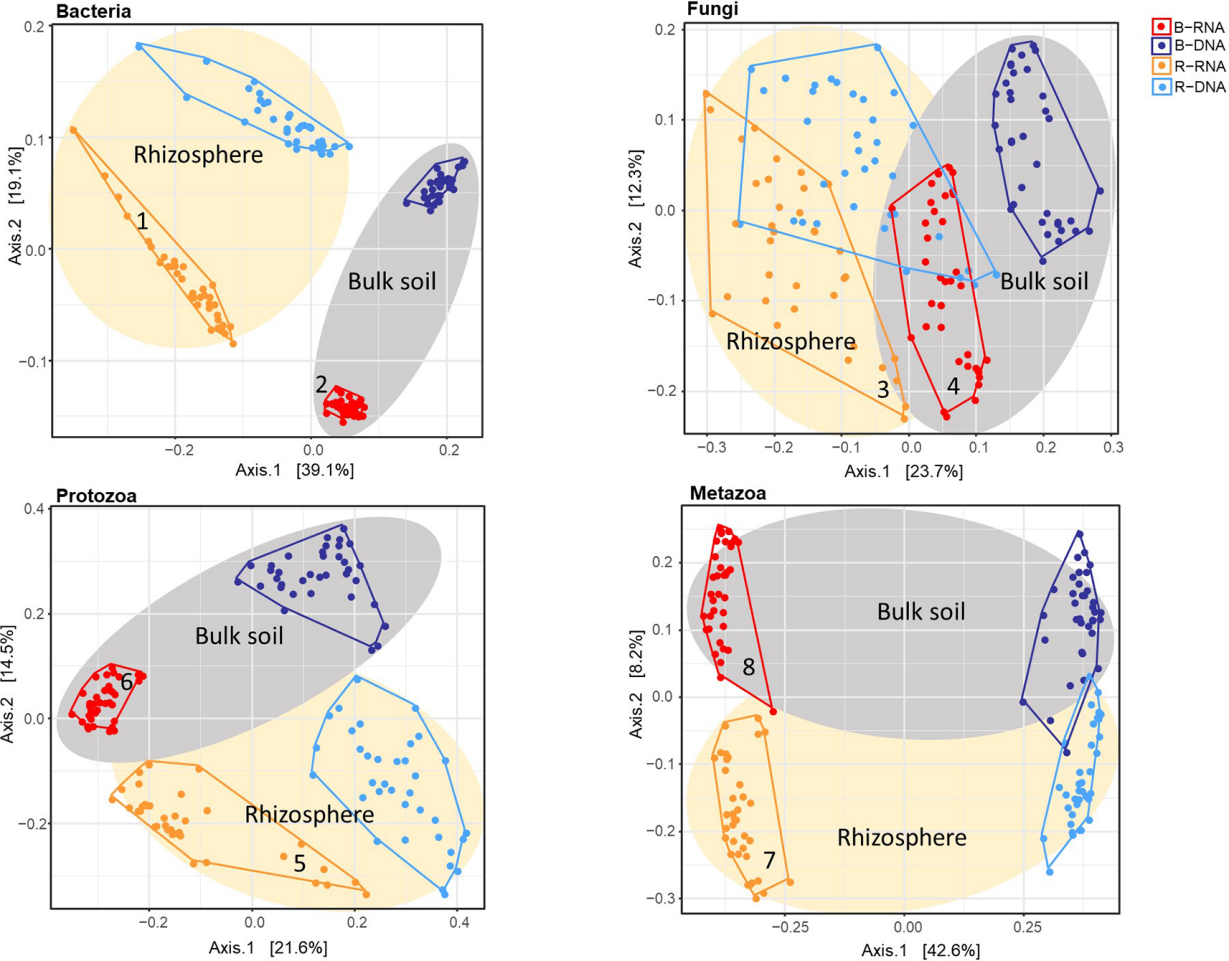
Principal coordinate analysis (PCoA) ordination of a Bray-Curtis dissimilarity matrix. Plots illustrate distances between communities [72 soil samples; for each sample both the resident (rDNA) and the active (rRNA) community were characterized] for each organismal group: **(A)** Bacteria; **(B)** Fungi; **(C)** Protozoa, and **(D)** Metazoa. Colours were used to distinguish between rRNA-bulk (red), rRNA-rhizosphere (ochre), rDNA-bulk (dark blue), and rDNA-rhizosphere (light blue). For all organismal groups: grey ellipses for bulk and ivory ellipses for rhizosphere. Numbers accompanying the active bacterial, fungal and protozoan communities (ochre and red) correspond to the numbers in the top left or top right corner of **Figure 2**.

To investigate whether contrasts could be observed between resident and active fractions of the individual organismal groups, and between bulk and pea rhizosphere soil, principal coordinate analysis (PCoA) ordinations on Bray-Curtis dissimilarity matrices were conducted (**Figure 1**). The effect of the variable Nucleic Acid (rRNA for active and rDNA for the resident community) is clearly visible. For all four organismal group there are distinct clusters for rRNA (red and ochre) and rDNA (blue and light blue) although this is somewhat less pronounced in case of Fungi. The effects of sample type (bulk *vs* rhizosphere) were easily observable as well. Especially for bacteria there is a clear separation of the two soil compartments (**Figure 1**).

To determine whether soil organismal groups were significantly affected by the four main variables included in this study three distinct methods to compare communities were used: Bray-Curtis dissimilarity, and weighted and unweighted UniFrac. Results of the PERMANOVAs are shown in **Table 1**. The R^2^ values indicate how much of the observed variation is explained by each of the individual variables. Sample Type (bulk *versus* rhizosphere soil) was the dominant explanatory variable for the observed shifts in the bacterial communities. The large difference between the relevant R^2^ values resulting from the Unifrac analyses (unweighted 16.4%, weighted 51.3%), points at a quantitative rather than a qualitative shift. For fungi, Sample Type was most important in the Bray Curtis analysis only. Phylogeny-based distance methods identified Nucleic Acid—the difference between the resident and the active fungal community—as the most dominant variable explaining 12.6 and 25.0% of the observed variations. For the protozoa and the metazoa, Nuclei Acid was identified as the main explanatory factor as well (except for the weighted Unifrac in case of Metazoa).

**Table 1:** The impact of four variables on four organismal groups in fields with three soil management regimes with pea as main crop.

	Bray-Curtis		UniFrac - Unweighted		UniFrac - Weighted	
**Bacteria**	**R^2^**	**P**	**R^2^**	**P**	**R^2^**	**P**
Nucleic Acid	0.231	0.001	0.137	0.001	0.261	0.001
Treatment	0.076	0.001	0.086	0.001	0.042	0.001
Sample Type	**0.295**	0.001	**0.164**	0.001	**0.513**	0.001
Time Point	0.040	0.001	0.025	0.001	0.021	0.001
Residuals	0.357		0.589		0.163	
**Fungi**						
Nucleic Acid	0.108	0.001	**0.126**	0.001	**0.250**	0.001
Treatment	0.134	0.001	0.102	0.001	0.091	0.001
Sample Type	**0.172**	0.001	0.066	0.001	0.177	0.001
Time Point	0.045	0.001	0.028	0.001	0.039	0.001
Residuals	0.542		0.679		0.443	
**Protozoa**						
Nucleic Acid	**0.192**	0.001	**0.305**	0.001	**0.434**	0.001
Treatment	0.082	0.001	0.088	0.001	0.047	0.002
Sample Type	0.125	0.001	0.099	0.001	0.106	0.001
Time Point	0.091	0.001	0.039	0.001	0.083	0.001
Residuals	0.510		0.469		0.329	
**Metazoa**						
Nucleic Acid	**0.440**	0.001	**0.104**	0.001	0.072	0.001
Treatment	0.040	0.001	0.089	0.001	0.046	0.001
Sample Type	0.063	0.001	0.100	0.001	**0.216**	0.001
Time Point	0.022	0.001	0.023	0.001	0.059	0.001
Residuals	0.440	0.001	0.104	0.001	0.072	0.001

Summary of the PERMANOVA for Bray-Curtis dissimilarity distances as well as phylogenetic distances (UniFrac). This analysis tests differences in quantitative taxonomic composition of Bacteria, Fungi, Protozoa and Metazoa assemblages taking Nucleic Acid (cDNA/DNA), Sample type (Bulk/Rhizosphere), Treatment (ConSlu, ConMin, Org) and Time point (Vegetative/Generative) as main variables. For each of organismal group and each analysis method the variable with the highest R^2^ is indicated in bold. Differences are considered significant if P < 0.01.

### Active Taxa Contributing to the Difference Between Bulk Soil and Rhizosphere

**Figure 2:**
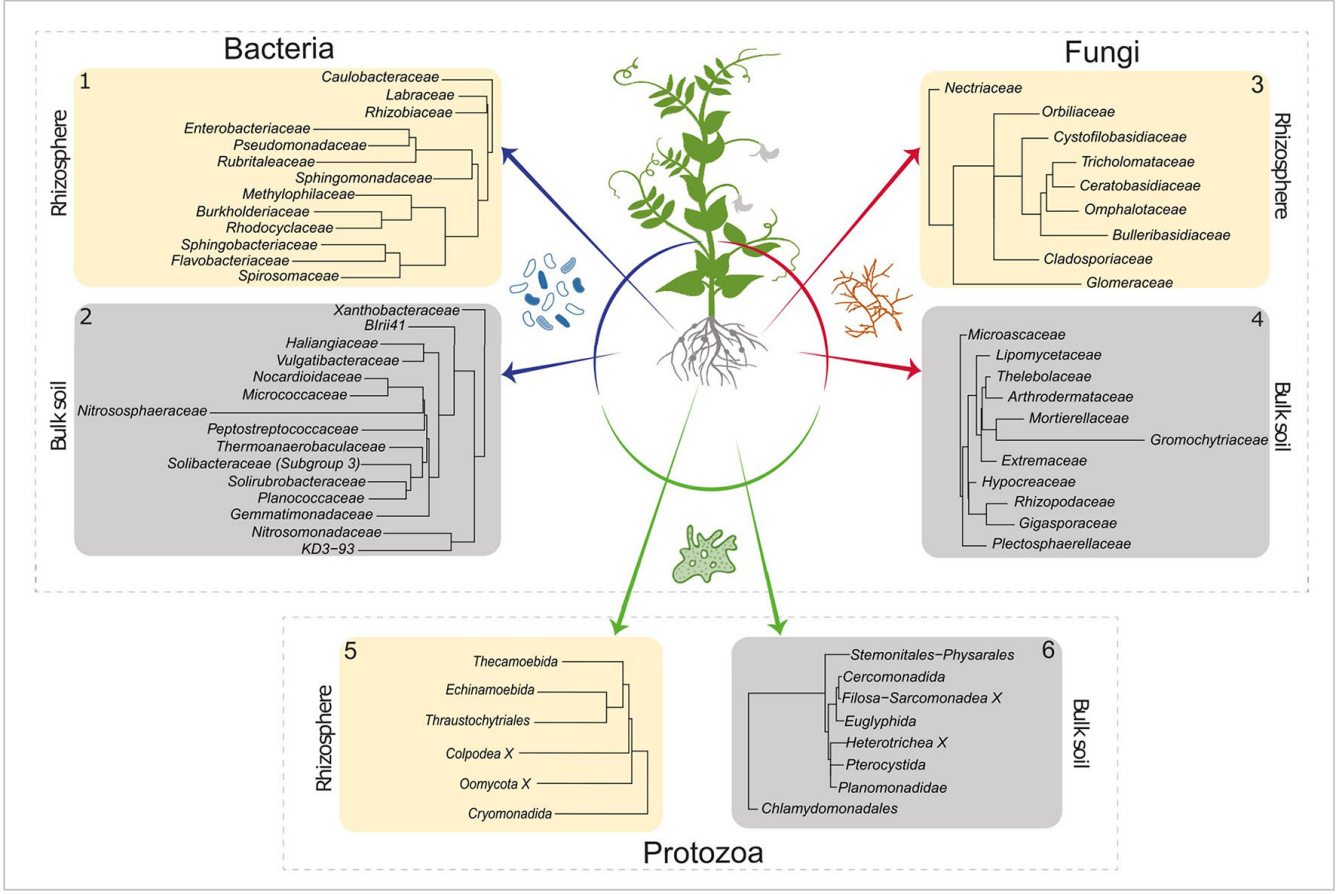
LEfSe analysis of the active bacterial, fungal, and protists OTUs. Identifying taxa for which a major part of the population was active in rhizosphere (ivory square) or in bulk soil (gray square) (LDA score > 2.5). Numbers in the top left or top right corner correspond to the number next to the active communities (ochre and red) in **Figure 1**. LEfSe, linear discriminate analysis effect size; OTUs, Operational Taxonomic Units; LDA, linear discriminate analysis.

LEfSe was used to determine which active taxa have the highest contribution to the observed differences between bulk and rhizosphere. An LDA threshold of >2.5 was set, which resulted in 28 bacterial, 20 fungal, and 14 protozoan orders that gave rise to the differences between bulk and rhizosphere communities (**Figure 2**). It is noted that metazoans are not displayed. Soil samples of 2 g were analysed, and the sample size is too low to give a genuine impression about the composition of the micro and mesofauna. Metazoa were nevertheless taken along as co-extraction of their DNA is indicative for spatial association of microbial taxa and the detected metazoans.

The elevated activities of members of Rhizobiaceae and Labraceae (both Rhizobiales) were detected in the rhizosphere of pea. As *P. sativum* is nodulated by the N_2_–fixing *Rhizobium leguminosarum*, an enrichment of the Rhizobiaceae was expected (**Figure 2**, panel 1). Moreover, increased activity was observed for number of bacterial families that harbour P-solubilizing members such as Rhizobiacea (including the genus *Rhizobium*), Enterobacteriaceae (including *Serratia*), Pseudomonadaceae (including *Pseudomonas*) and Burkholderiaceae (including *Burkholderia*) (Pii et al., 2015). In the bulk soil we observed a relatively high activity of the Nitrosophaeroceae (Archaea, Thaumarchaeota). Representatives of this family are known as ammonia oxidizers (Stieglmeier et al., 2014). In addition, a bacterial family that harbours members that initiate the oxidation of ammonia to nitrite, the Nitrosomonadaceae, showed enhanced activity. Regarding fungal families with upregulated activity in the rhizosphere at least two observation are noteworthy (**Figure 2**, panel 3). The Orbiliaceae harbour numerous nematophagous fungi, and this could affect the RKN *Meloidogyne chitwoodi* present in these experimental fields (Xie et al., 2010). Differential activity of two members of the Glomeromycota were observed in the two compartments. Whereas Glomeraceae (order Glomerales) showed enhanced activity in the rhizosphere, elevated levels of Gigasporaceae (order Diversisporales) activity were detected in the bulk soil (**Figure 2**, panels 3 and 4). This observation suggests that pea might interact with a member of the order Glomerales.

Members of the order Thecamoebida (**Figure 2**, panel 5) are known as large protists and voracious predators of bacteria and other protozoans (Melton et al., 2019), which explains their elevated activity in the rhizosphere. Another representative of the naked amoebae, the Echinamoebida, was highly active in the rhizosphere. Terrestrial Colpodea, small bacterivorous ciliates, are known as extreme r-strategists (Lüftenegger et al., 1985), and they can easily cope with fluctuating environmental dynamics, this might explain their enhanced activity in the rhizosphere (Foissner, 1993). The detection of active Pterocystida in bulk soil (**Figure 2**, panel 6) is remarkable as they belong to the heliozoan protists that are normally found in freshwater and marine environments, and only occasionally in soil (Cavalier-Smith and Chao, 2012). Also, the enhanced presence of active Chlamydomonadales, an order of green algae, is worth noting. We assume that these photosynthesizing protists were present at the very top layer of the bulk soil.

### Difference Between Resident and Active Communities Under Three Distinct Soil Management Regimes at Organismal Group Level

To determine the level at which the soil management regimes ConMin, ConSlu, and Org had an effect on the four organismal groups, we analysed the rDNA sequence data separate from the rRNA data. As can be seen in **Table 2**, soil management (“Treatment”) had a significant effect on both the composition of the communities, and their levels of activity for all four organismal groups (in all cases P< 0.001). These analyses also showed that the compartment effect, the contrast between rhizosphere or bulk soil, is consistently larger than the treatment effect.

**Table 2:** The impact of three variables on the resident and the active fractions of four organismal groups in fields with three soil management regimes with pea as main crop.

	rDNA		rRNA	
**Source**	**R^2^**	**P**	**R^2^**	**P**
**Bacteria**				
Treatment	0.101	0.001	0.103	0.001
Sample Type	**0.378**	0.001	**0.428**	0.001
Time Point	0.048	0.001	0.063	0.001
Residuals	0.472		0.406	
**Fungi**				
Treatment	0.154	0.001	0.165	0.001
Sample Type	**0.218**	0.001	**0.208**	0.001
Time Point	0.047	0.001	0.063	0.001
Residuals	0.581		0.565	
**Protozoa**				
Treatment	0.139	0.001	0.132	0.001
Sample Type	**0.241**	0.001	**0.159**	0.001
Time Point	0.136	0.001	0.120	0.001
Residuals	0.484		0.589	
**Metazoa**				
Treatment	0.138	0.001	0.101	0.001
Sample Type	**0.168**	0.001	**0.200**	0.001
Time Point	0.064	0.001	0.067	0.001
Residuals	0.630		0.632	

This summary of the PERMANOVA for Bray-Curtis dissimilarity distances shows the impact of Treatment (ConSlu, ConMin, Org), Sample type (Bulk/Rhizosphere), and Time point (Vegetative/Generative) on four categories of soil inhabitants. For each of organismal group and each analysis method the variable with the highest R^2^ is indicated in bold. Differences are considered significant if P <0.01. For Unifrac analyses see **Supplementary Table S2**.

**Figure 3:**
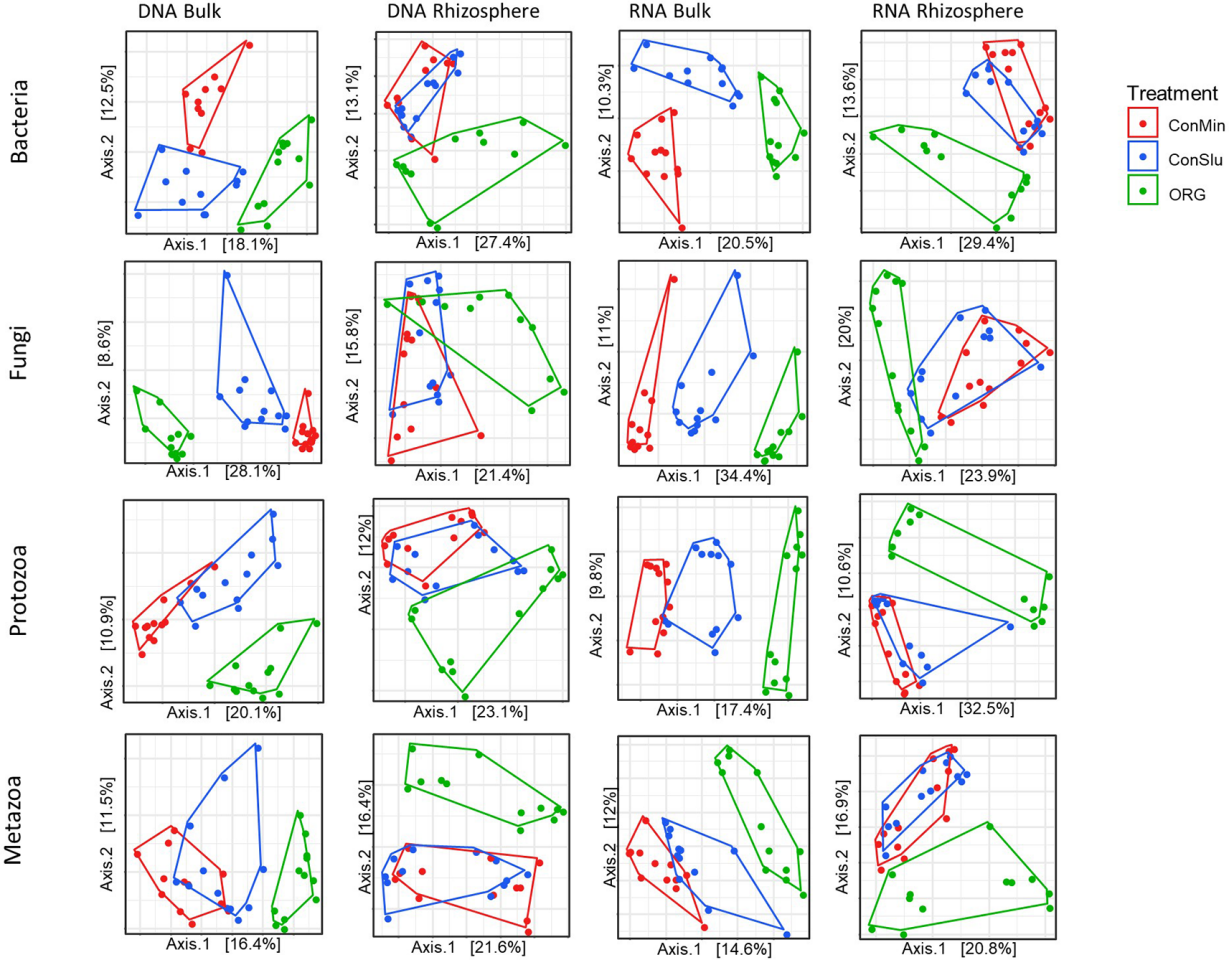
Principal coordinate analysis (PCoA) ordination of a Bray-Curtis dissimilarity matrix. Plots illustrate distances between communities for each of the organismal groups under three different soil management types [ConMin (red), ConSlu (blue), and Organic (green)]. Split plots per organismal group for DNA bulk, DNA rhizosphere, RNA bulk, and RNA rhizosphere.

Principal coordinates analysis (PCoA) was used to visualize the effect of prolonged exposure to three distinct management regimes in bulk soil and rhizosphere on the active and resident communities of each organismal groups. As can be seen in **Figure 3**, soil management had a major impact on all four organismal groups. ‘DNA bulk’ shows the resident communities in absence of the main crop, and the communities in the fields under organic management were distinct from two conventional treatments, ConMin and ConSlu. In case of ‘RNA bulk’, the same trend was observed. Another interesting shift was observed within the two conventional treatments (ConMin in red and ConSlu in blue). Whereas the soil communities in bulk soil were fully separated in all three different management types, the two conventional treatments tend to overlap in their communities in the rhizosphere. It is noted that the highest percentage of the variation explained by the two axes was observed for “RNA rhizosphere” with an average of 44.4% for the four organismal groups.

### Active Rhizosphere Taxa Contributing Most to the Observed Difference Between the Three Soil Management Regimes

**Figure 4:**
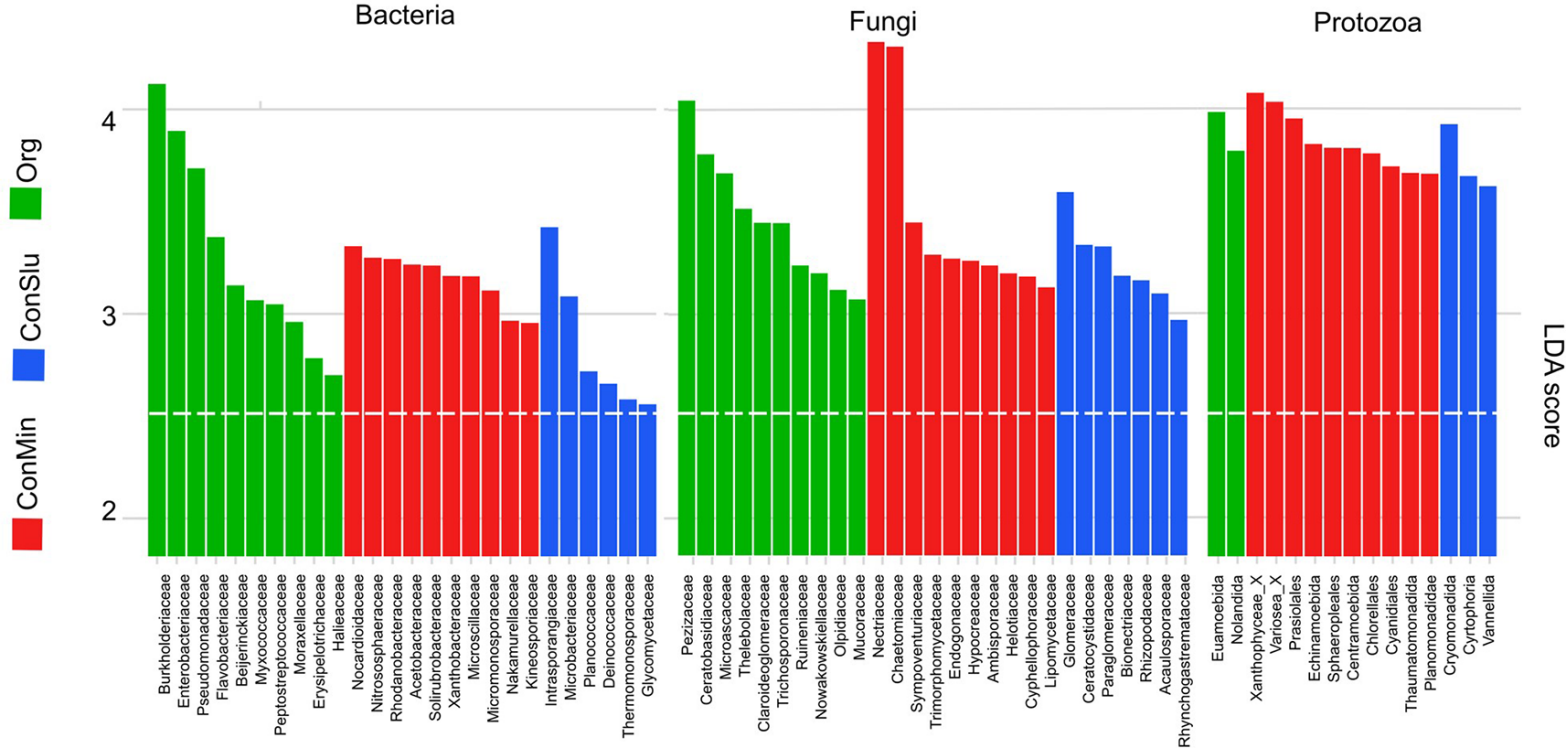
Discriminant active bacterial, fungal, and protozoan taxa in the rhizosphere indicated by LEfSe analysis (LDA threshold of 2.5) resulting from distinct soil management types at location Vredepeel: ConMin (red), ConSlu (blue), and Org (green). LEfSe, linear discriminate analysis effect size; LDA, linear discriminate analysis.

**Figure 4** shows the results of LEfSe analyses that revealed the taxa that contributed most to observed differences in the active pea rhizosphere communities under the three soil management systems. Regarding the bacterial community, families harbouring P-solubilizing members such as the Burkholderiaceae, the Enterobacteriaceae, and the Pseudomonadaceae showed high levels of activity in the organic fields. Members of the Nocardioidaceae showed elevated levels of activity under the ConMin regimes, the family was recently identified as being associated with the domestication of a pea relative, the common bean *Phaseolus vulgaris* (Perez-Jaramillo et al., 2017).

The Pezizaceae stood out as being active under the organic soil management regime. This fungal family harbours dozens of genera, and often grow on dung of animals. As the organic plots only received farmyard manure and cow slurry, the enrichment of this speciose fungal family can be seen in this perspective (Alexopoulos and Mims, 1979). Both Actinobacterial families, Intrasporangiaceae and Nocardioidaceae, showing a high level of activity in the conventional soil treatments ConSlu and ConMin (**Figure 4**). Both families are known as efficient secondary utilizers of cellulose-derived glucose under oxic conditions (Schellenberger et al., 2010). This observation points at enhanced cellulolytic activity in the pea rhizosphere under conventional soil management.

The families Nectriaceae and Chaetomiaceae were identified as fungal indicators for fields with the most conventional treatment (ConMin). The high activity of Nectriaceae might be related to the application of mineral fertilizer. A similar phenomenon was observed in tropical rain forest plots treated with mineral fertilizer (Kerekes et al., 2013). The other upregulated member of the Sordariales, the family Chaetomiaceae, is known for its cellulolytic members (Wilhelm et al., 2017). This enhanced activity might have facilitated the elevated activity of members of the Intrasporangiaceae and Nocardioidaceae.

The Tubulinea orders Euamoebida and Nolandida were metabolically active in the organic fields. The first family was found in high relative abundances in grasslands and forest mineral soils, whereas the Nolandida is a relatively rare protist order in soils (Geisen et al., 2015). Xantophyceae, consisting of stramenopilan photoautotrophs, typify the ConMin fields. Other phototrophs showing enhanced activity in the ConMin fields were members of the green algae order Chlorellales, and the red algae order Cyanidiales.

### Soil Management-Related Shifts in Nematode Communities

**Figure 5:**
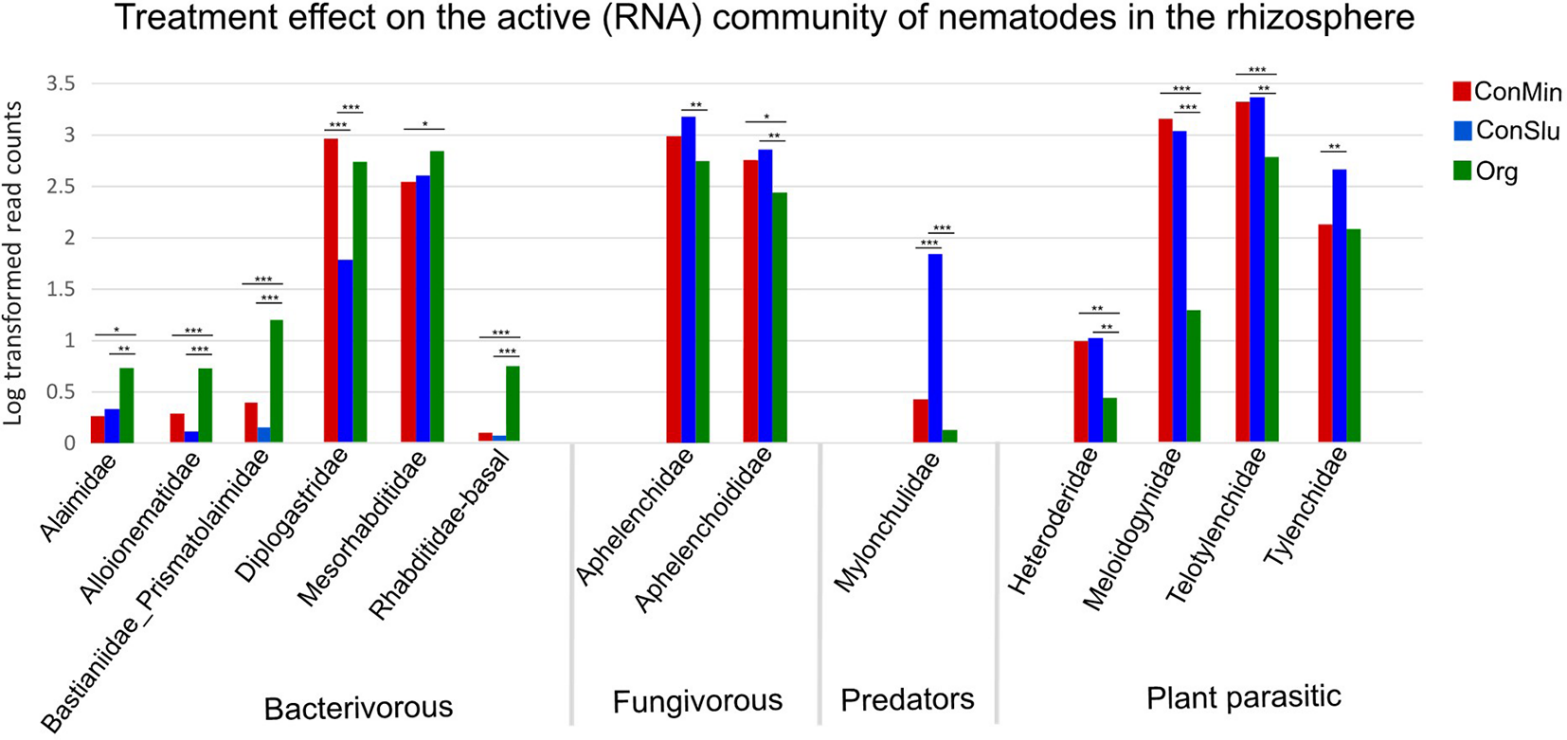
Shifts in the active nematode communities in the rhizosphere for each of the three soil management regimes: ConMin (red), ConSlu (blue) and Org (green). Each bar represents the average of 36 data points (18 subplots were sampled at two time points). Kruskal-Wallis test, followed by Dunn’s Test. (* = p < 0.05, ** = p <0.01, *** = p <0.001).

Microscopic nematode density counts, and qPCR nematode density data showed no significant differences in overall nematode abundances between the three soil management types (**Table 3**). By means of rRNA sequencing, taxonomic shifts in nematode communities were detected at family level (**Figure 5**). Five out of six bacterivorous families were found to be more abundant in Org fields. In case of the two fungivorous families Aphelenchidae and Aphelenchoididae, small but significant trends towards lower densities in fields under organic management were observed. The predator family Mylonchulidae was specifically upregulated in the ConSlu fields. With regard to the plant parasites, lower infestation levels for the families Telotylenchidae, Heteroderidae, and Meloidogynidae were observed in the organic fields.

**Table 3:** Mean nematode densities and *M. chitwoodi* abundances (individuals per 100g soil) in the pea fields with three distinct soil management regimes.

	**ConMin**	**ConSlu**	**Org**
**Nematode density** (m)	3100	3210	2880
**Nematode density** (q)	1551	1622	1440
***M. chitwoodi*** (q)	58^a^	65^a^	2^b^

Significant differences are indicated by lowercase a or b (Post-hoc Bonferroni P < 0.05). Samples were analyzed microscopically (m) or with quantitative PCR (q).

MiSeq nematode community analysis does not allow for the detection of nematodes at species level, and therefore a species-specific qPCR assay was run to pinpoint the observed decrease of members of the genus *Meloidogyne.* qPCR analysis revealed that the *Meloidogyne chitwoodi* were significantly lower in the Org fields as compared to the two conventional treatments (P< 0.05). Soil samples were checked for the presence of other *Meloidogyne* species (**Supplementary Table S1**), and these were not present or at very low levels only. Therefore, we conclude that the observed difference in Meloidogynidae levels in **Figure 5** (based on rRNA data) can be attributed predominantly to the Columbian RKN *M. chitwoodi*.

Commonly used nematode extraction protocols exploit the mobility of nematodes to separate the roundworms from the soil matrix. Hence, non-active nematodes will not be extracted by these methods. Direct extraction of nematode DNA and RNA from soil does not include this selection step, and therefore we compared rRNA-based results (**Figure 5**) with results with rDNA (**Supplementary Figure S4**). Both figures show significant lower abundances of the monogeneric family Meloidogynidae in the organic fields. Hence, all three approaches, rDNA or rRNA-based sequencing and species-specific qPCR assays, demonstrate significantly lower RKN levels in Org fields.

## Discussion

Mapping of resident and active fractions of the primary decomposer community—bacteria and fungi—as well two major primary consumer groups—protists and nematodes—under three distinct soil management regimes revealed that pea exerts a large effect on the soil microbiome. Below we will discuss (1) how the current characterisation of the pea rhizobiome relates to other studies, (2) how our observations regarding the effect of soil management relate to previous findings, and (3) whether we can find plausible biological explanations for the observed sharp decline in RKN densities in fields under prolonged organic soil management.

### How Does the Current Characterisation of the Pea Rhizobiome Relate to Previous Studies?

As exemplified by the impact of pea, lentil and chickpea, legumes have been shown to exert a large influence on the soil microbiome as compared to other crops such as cereals (Turner et al., 2013; Hamel et al., 2018). N rhizodeposition has been shown to comprise 13% of the total plant N for pea (Mayer et al., 2003), and presumably this has contributed to this large impact. The large overall effect of legumes could be corroborated by comparing the current study with a recent study on the barley rhizobiome that made use of the same experimental fields (Harkes et al., 2019). In case of barley, the compartment effect (bulk *vs* rhizosphere) explained a smaller percentage of the observed variation than impact of the soil management regime. For pea, on the contrary, all four organismal groups indicated the compartment effect to be larger than the soil treatment effect. Hence, under similar experimental conditions the compartment effect on the soil microbiome induced by pea (a legume) is stronger than the effect induced by barley (a cereal).

Recently, the effect of various frequencies pulse crop cultivation (including pea) on resident soil bacterial communities was mapped (Hamel et al., 2018). Increased frequency of pulse cultivation resulted in higher abundances of α-Proteobacteria in the rhizosphere, and a decrease in γ-Proteobacteria (although the latter was accompanied by an increased presence of *Pseudomonas*). Keeping in mind that the Rhizobiales (in our study Rhizobiaceae and Labraceae) belong to the subclass α-Proteobacteria, an overall increase of this subclass was to be anticipated. Moreover, Hamel et al. (2018) detected an increase *Pseudomonas* read in rotations involving pea. This might correspond to the increased activity of Pseudomonadaceae we observed in the pea rhizosphere (**Figure 2**, panel 1). We could not confirm the increased presence of Actinobacteria in the pea rhizosphere as observed in rotation systems with frequent inclusion of pulses (referred to as “3-pulse systems”). This phenomenon might only be observable after repeated cultivation of legumes.

We conclude that a number of parallels can be discerned between studies on the effect of pea on the rhizobiome. It is noted that differences in experimental approach (focus on resident or active soil biota) and set up (soil type, soil management practices) complicates the identification of generic effects of legumes on the soil living community.

### How Does the Current Characterisation of the Effects of Soil Management on the Soil Microbiome Relate Other Studies?

In a long-term (>10 years) greenhouse experiment the effect of organic, integrated and conventional farming systems on the soil rhizobiome was investigated (Li et al., 2019). The authors identified a bacterial hub, a small number of highly interconnected taxa, consisting of *Bacillus* (Bacillaceae), *Sporosarcina* (Planococcaceae), *Hyphomicrobium* (Hyphomicrobiaceae), *Gaiella* (Gaiellaceae), as well as *Pirellula* and *Blastopirellula* (both Planctomycetaceae) that were significantly more abundant in soil from the organic management regime (Li et al., 2019). Another hub comprising of the genera *Rhizobium* (Rhizobiaceae), *Sphingobium* (Sphingomonadaceae), *Pseudoxanthomonas* (Xanthomonadaceae), and *Dyadobacter* (Cytophagaceae) was present in higher densities in the conventional and the integrated treatments. These findings show very little resemblance with the bacterial taxa that were shown to be activated under the organic or one of the two conventional soil managements systems in the present study (**Figure 4**). From this, we conclude that the plant effects can be stronger than the effect of soil management (variable “treatment” in **Tables 1** and **2**). Moreover, it is noted that the active bacterial community can be quite distinct from the resident bacterial communities mapped by Li et al. (2019) (**Figure 1**).

In another long-term field experiment (running for ≈ 15 years at time of sampling) fields were continuously exposed to either conventional or organic farming practices, and the impact of the practices on bulk soil were determined (Bakker et al., 2018). These authors showed a remarkable contrast between bacterial phyla with regard to the extent by which they were affected by the contrasting farming practices. More taxa showed higher abundances in organic as compared to conventional farming. Moreover, some bacterial phyla such as Chloroflexi, Firmicutes, and Gemmatimonadetes seemed to be unaffected by farming practice while others such as Proteobacteria, Acidobacteria, and Verrucomicrobia were. Our data only partly support this observation. The families Burkholderiaceae and Hyphomicrobiaceae (Proteobacteria) and the Peptostreptococcaceae (Firmicutes) were both more abundant and more active in bulk soil in organic fields (**Supplementary Figure S3**). Peptostreptococcaceae are one of the dominant family in the gut microbiome of earthworms (see *e.g.* Zeibich et al., 2018), and as such this result could point at an elevated presence of earthworms in fields under organic management. Our analysis of resident bacterial community in bulk soil under the organic regime, also showed an increase of members of the Acidobacterial family Blastocatellaceae (**Supplementary Figure S3**). In the most conventional soil management system (ConMin), the Verrucobacterial family Pedosphaeraceae was both abundant and highly activated. At high taxonomic level, this is in line with the observations presented by Bakker et al. (2018).

Hence, despite the fact that plant identity may have a stronger effect on the rhizobiome than soil management practices, the effect of these practices could be pinpointed at taxon level. Our data suggest that the working hypothesis saying that only a subset of the soil bacterial phyla is affected by conventional or organic soil management practices might be correct.

### Can We Pinpoint Nematode-Suppressive Bacterial or Fungal Taxa That Might Underlie the *M. chitwoodi* Decline in Fields Under Organic Management?

In this study we investigated the soil microbiome of pea in fields naturally infested with *M. chitwoodi* under three different soil management regimes, conventional, integrated, and organic. *M. chitwoodi* is a highly polyphagous plant parasite infecting numerous mono- and dicotyledonous crops, including pea (*P. sativum*) (Oepp/Eppo, 1991), and it has a reputation as a major pest in potato. In all studied fields here, *M. chitwoodi* was already present for multiple years (Visser et al., 2014). As potato—a highly suitable host—was the main crop in the previous growing season, we expected the *M. chitwoodi* population to be physiologically fit at the onset of the pea growing season.

At the end of the growing season, *M. chitwoodi* densities in the two conventional soil management systems harbours ≈ 60 individuals per 100 g soil, whereas about two individuals per 100 g were detected in the organically managed system. We investigated whether a biological explanation could be found for this difference. Within the bacterial and fungal rhizosphere communities, families were detected that are known to harbor multiple nematode-trapping species. As shown in **Figure 2** (panel 3), the Orbiliaceae were shown to be active in the pea rhizosphere. This family comprises genera such as *Arthrobotrys*, *Dactylella*, and *Monacrosporium.* These genera are essentially saprophytic fungi but can become predatory under *e.g.* low nutrient conditions (Gray, 1987). As a response, the fungi will form traps (*e.g.* constricting rings, adhesive networks) which allow them to prey on nematodes (Xie et al., 2010). We verified whether Orbiliaceae activity was upregulated in the fields under organic management. This was not the case, and even a non-significant trend towards lower activity in organic fields was observed (**Supplementary Figure S2**). Elevated activity of another fungal family, the Olpidiaceae, typified fields under organic soil management. A member of this family, *Olpidium vermicola*, has been reported to parasitize eggs and females of endoparasitic nematodes such as *M. chitwoodi* (Esser and Schubert, 1983; Askary, 2015). However, other *Olpidium* species are virus-transmitting plant pathogens. Zoospores of *Olpidium virulentus* colonize roots of various plant species, and were demonstrated to accumulate in crop rotation with multiple pulses including pea (Niu et al., 2018).

The bacterial family Pseudomonadaceae was also identified as an indicator species for organic farming (**Figure 4**). Further analyses identified the genus *Pseudomonas* as main contributor to the indicator status of Pseudomonadaceae. The *Pseudomonas* species *P. aeruginosa, P. fluorescens, P. protegens, and P. chlororaphis* belong to ecologically most relevant nematode-suppressive bacteria in soil (Li et al., 2014). *Pseudomonas* species produce toxins which may inhibit hatching, survival and *M. chitwoodi*’s ability to penetrate plant roots (Thiyagarajan and Kuppusamy, 2014; Nandi et al., 2015; Kang et al., 2018).

The increased densities of bacterivorous nematode families might form an indirect explanation for the decrease of *M. chitwoodi* in organic soil management systems (**Figure 5**). As bacteria-grazing nematodes in the immediate vicinity of plant roots could locally improve nutrient availability *via* the excretion of easily uptakeable N and P. Plants could benefit from this in terms of improved growth and vitality, possibly making them less susceptible to plant-parasitic nematodes (Thoden et al., 2011).

Presumably multiple factors have contributed to significantly lower *M. chitwoodi* levels in the organic fields. This might have included nematode parasitic members of fungal genus *Olpidium* and/or the elevated activity of members of the Pseudomonadaceae. These results should be seen as potential leads for more detailed studies on the effect soil management regimes on the activity levels of nematode-suppressive bacteria and/or fungi.

### Concluding Remarks

The development of a time-efficient and affordable protocol to extract total DNA and RNA from soil (Harkes et al., 2019) allowed us to monitor the effect of a legume, pea, on both resident and active communities of primary decomposers as well as primary consumers of bacterial and fungal assemblages. Pea was shown to exert a large effect on the rhizobiome, and this was not only true for the primary decomposers but also for the protist and metazoan community. For all four organismal groups, and irrespective of the algorithm used to assess community shifts, the variables “Nucleic Acid” and “Sample Type”—representing respectively the differences between the resident and the active communities, and the effect of pea on the rhizobiome—had the highest impact on the soil microbiome. Notwithstanding this conclusion, soil management (“Treatment”) had also a significant effect on both the primary decomposers and the two primary consumer groups. A number of taxonomic groups (mostly at family level) were identified as contributors to these contrasts. In some cases, these taxa could be linked to treatment or crop identity, but in other cases such families were highly speciose or barely characterized from a soil ecological point of view. In essence, this was also true regarding our efforts to find possible biological explanations for the remarkably low levels of the RKN *M. chitwoodi* under the prolonged organic management regime. Our data suggest that Pseudomonadaceae—here members of the genus *Pseudomonas*—could have played a role in the biological suppression of this notorious RKN species. It should be underlined that biological associations have been identified in this research, and it was by no means proven that one or more *Pseudomonas* species were actually responsible for the observed decline in RKN levels in fields under organic soil management.

In this study a broad approach was used to characterize shifts in the soil microbial community under various soil management regimes with a legume—pea—as main crop. In our analyses we mainly focused on the active fractions, and this allowed us to pinpoint target taxa associated with the various treatments for each of the four organismal groups. One of the main remaining hurdles for the fundamental understanding of shifts in soil microbial communities is our fragmented and often poor knowledge about the ecologies of soil inhabitants.

## Data Availability Statement

The datasets generated for this study can be found in the: The raw sequences were submitted to the NCBI Sequence Read Archive (SRA) database under study accession numbers PRJNA561075 for bacteria, and PRJNA561072 for fungi, protozoa and metazoa.

## Author Contributions

PH, AS, JHa and JHe were responsible for the experimental design. PH, SE, AS, and MH were involved in sampling the rhizosphere. PH performed RNA/DNA isolation of the soil samples. PH performed the two step PCR reactions in order to prepare the sequence library. MH sorted the sequence data and analysed the nematode sequences. JS analysed the sequence data and performed the bioinformatics and statistical analysis. PH performed the statistical analyses on the nematode sequence data. PH and JHe wrote the manuscript; all others co-commented on the manuscript.

## Funding

All sources of funding received for this research have been submitted. This research was supported by NWO Groen grants (numbers: 870.15.021 and 870.15.022).

## Conflict of Interest

The authors declare that the research was conducted in the absence of any commercial or financial relationships that could be construed as a potential conflict of interest.
