# Supplementary material for: Shifts in the Active Rhizobiome Paralleling Low Meloidogyne chitwoodi Densities in Fields Under Prolonged Organic Soil Management
Source: Front Plant Sci. 2020 Jan 10;10:1697. doi: 10.3389/fpls.2019.01697 (PMC6965313; doi:10.3389/fpls.2019.01697)
Supplement: Supplementary file 1 [file Presentation_1.pptx]

## Slide 1
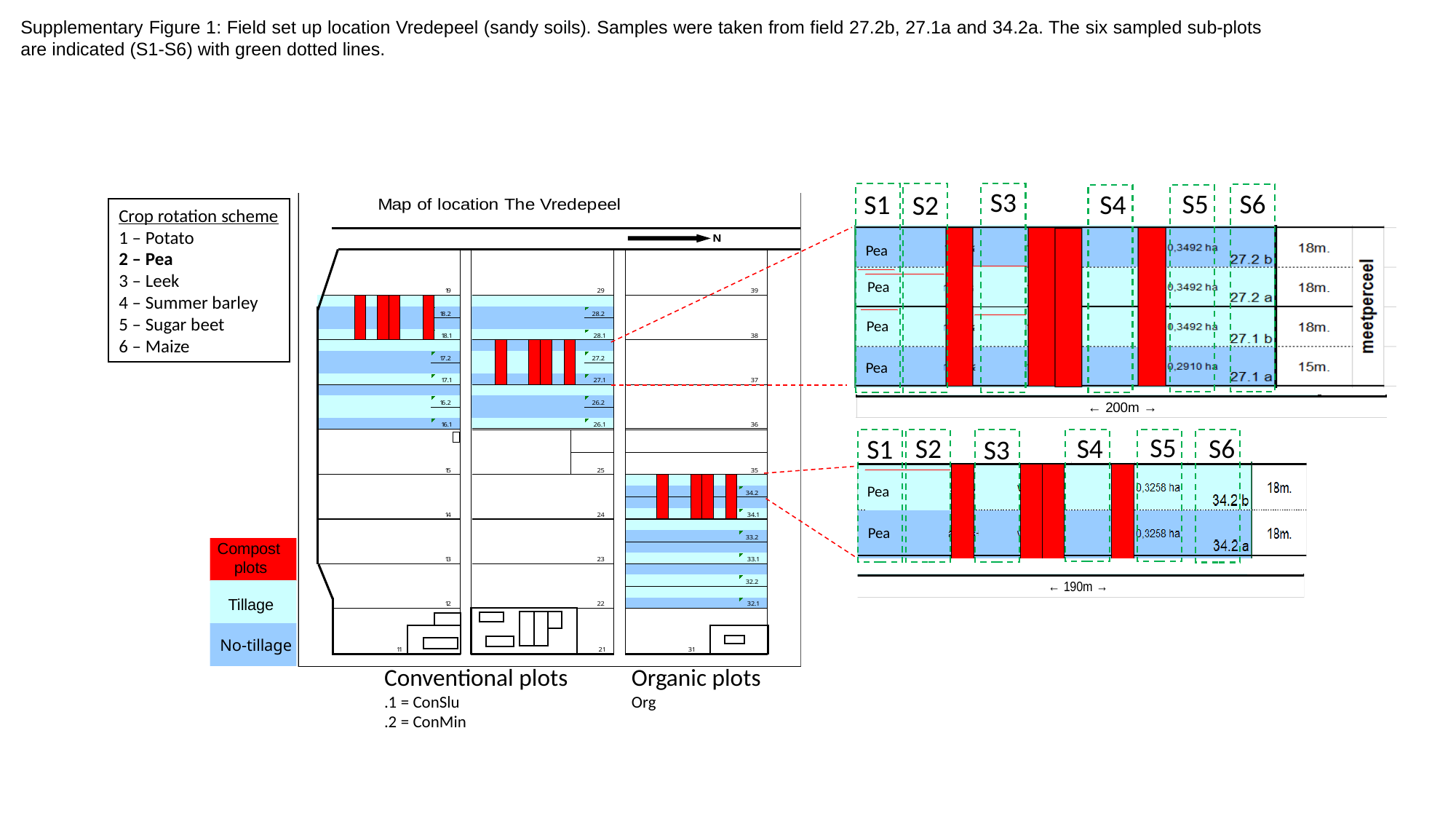

Supplementary Figure 1: Field set up location Vredepeel (sandy soils). Samples were taken from field 27.2b, 27.1a and 34.2a. The six sampled sub-plots are indicated (S1-S6) with green dotted lines.
S3
S5
S6
S1
S4
S2
Crop rotation scheme
1 – Potato
2 – Pea
3 – Leek
4 – Summer barley
5 – Sugar beet
6 – Maize
Pea
Pea
Pea
Pea
S5
S2
S6
S4
S1
S3
Pea
Pea
Compost
plots
Tillage
No-tillage
Organic plots
Org
Conventional plots
.1 = ConSlu
.2 = ConMin

## Slide 2
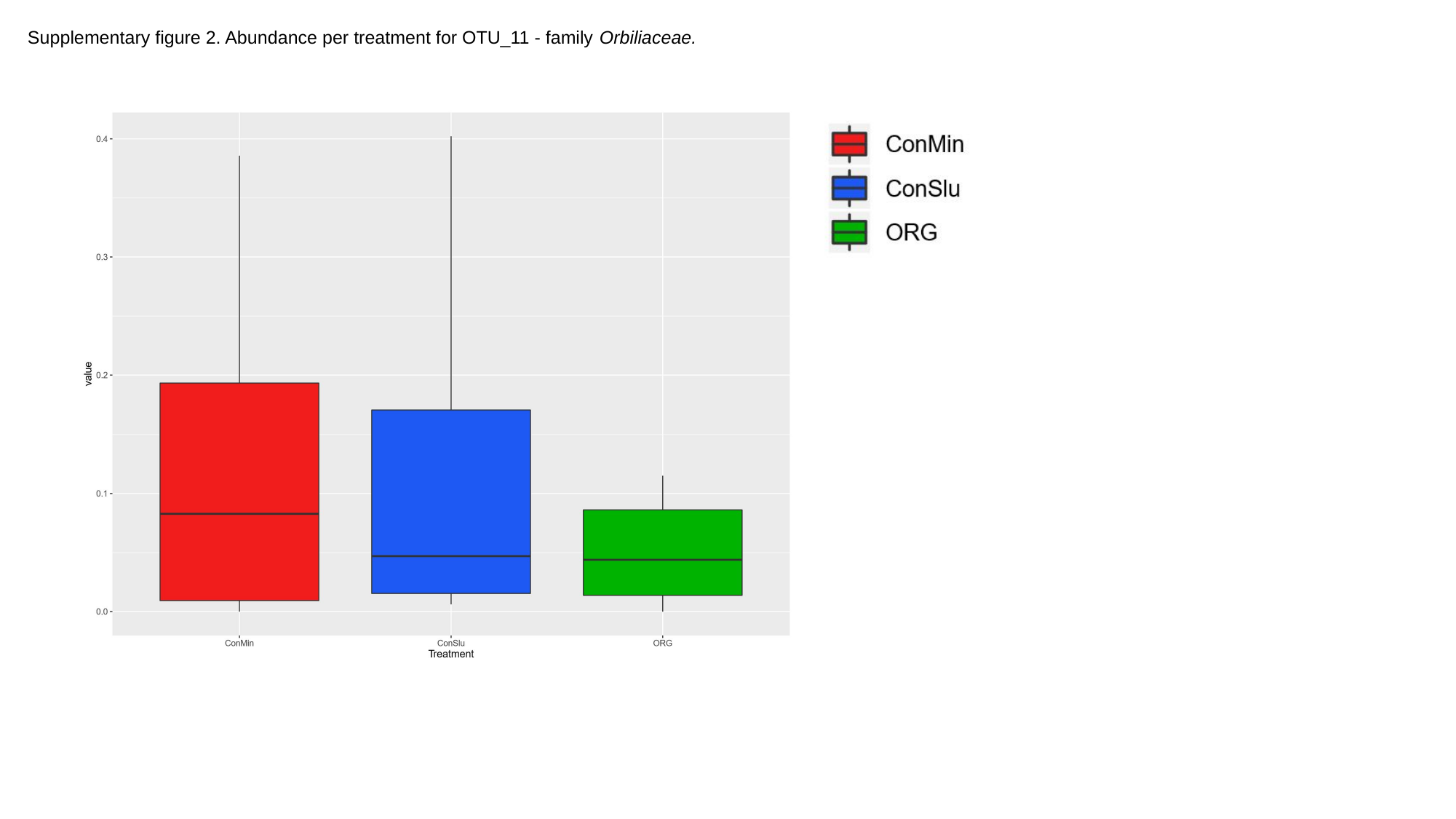

# Supplementary figure 2. Abundance per treatment for OTU_11 - family Orbiliaceae.

## Slide 3
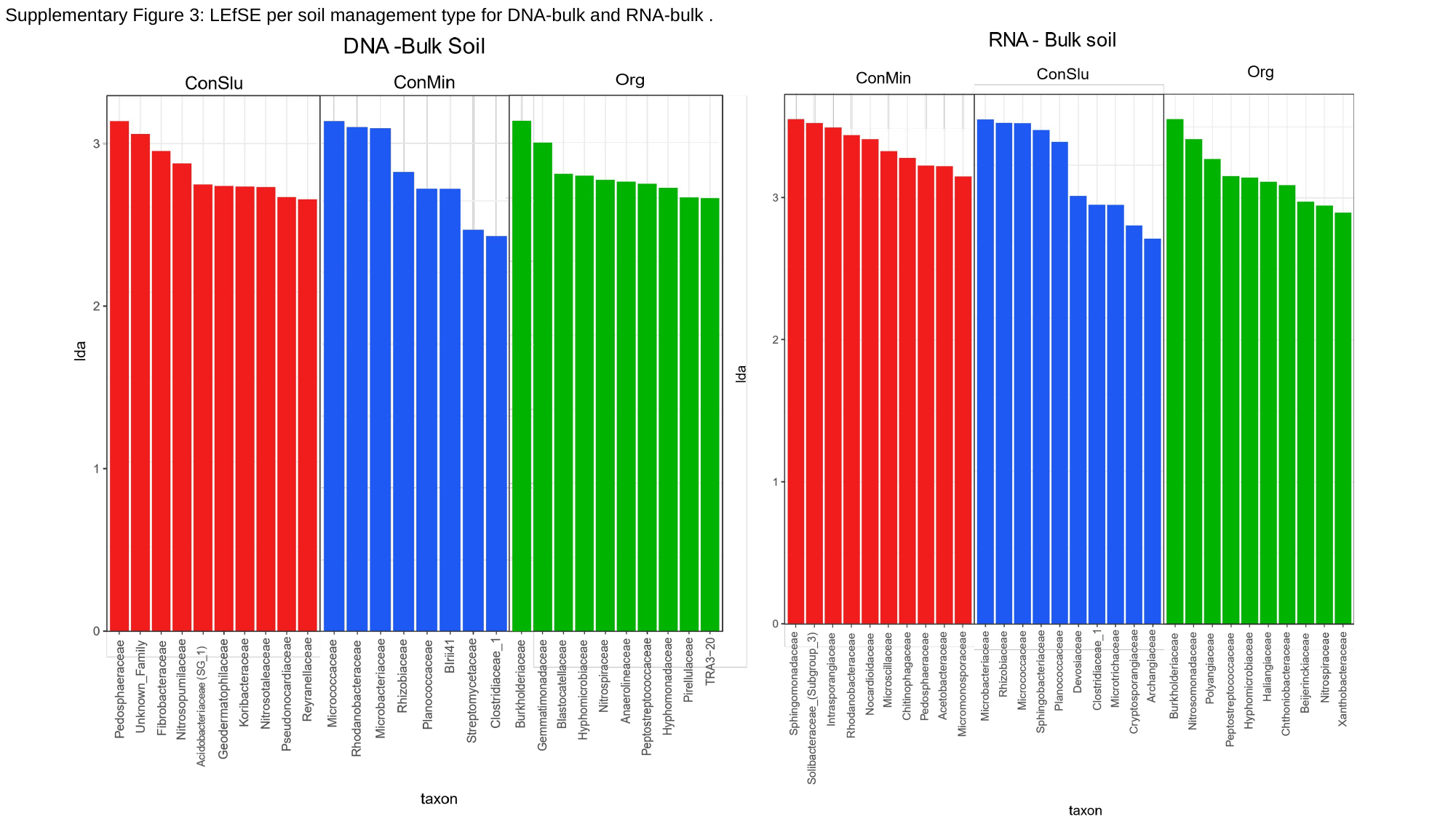

Supplementary Figure 3: LEfSE per soil management type for DNA-bulk and RNA-bulk .

## Slide 4
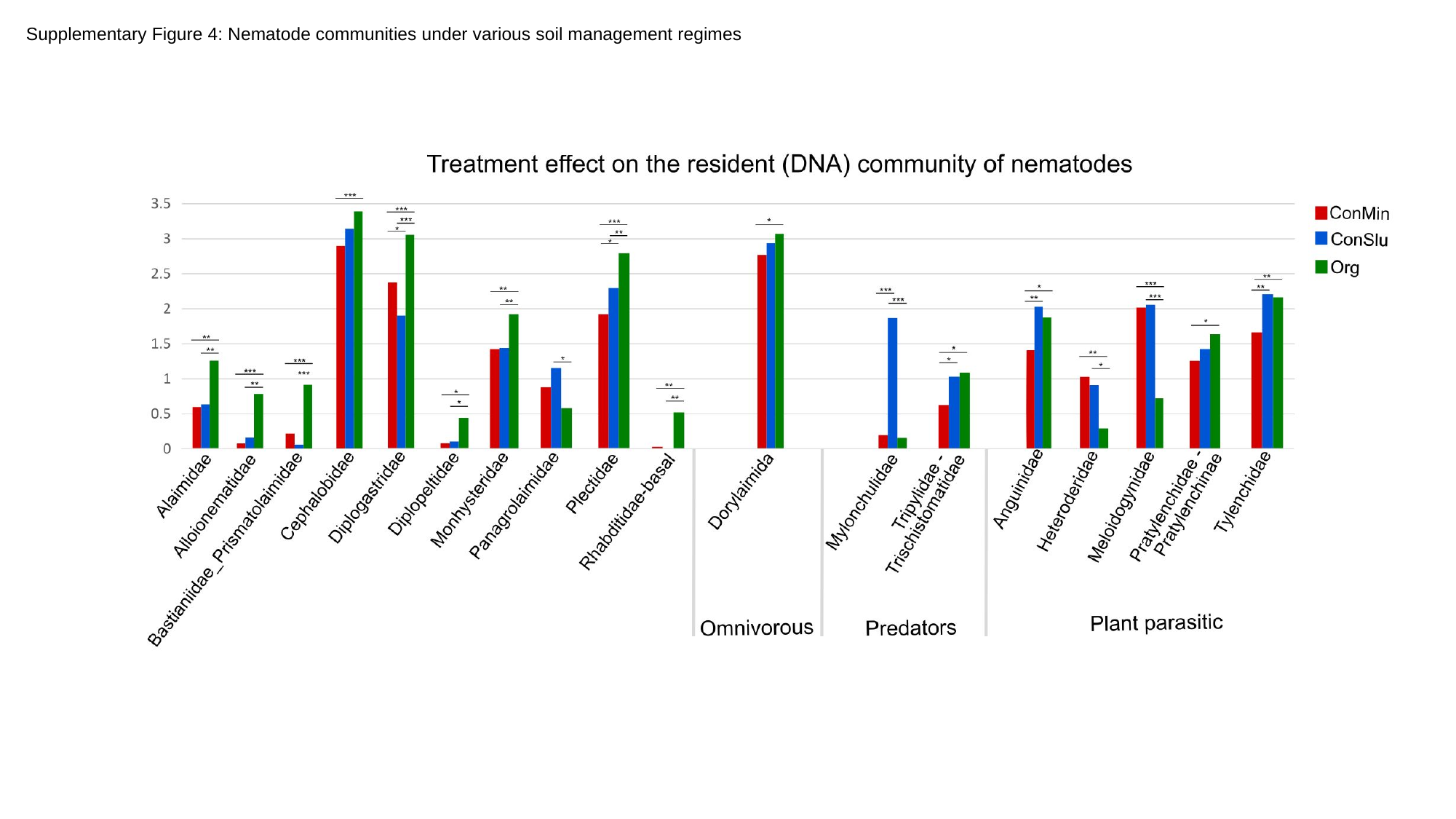

Supplementary Figure 4: Nematode communities under various soil management regimes
