# Supplementary material for: Shifts in the Active Rhizobiome Paralleling Low Meloidogyne chitwoodi Densities in Fields Under Prolonged Organic Soil Management
Source: Front Plant Sci. 2020 Jan 10;10:1697. doi: 10.3389/fpls.2019.01697 (PMC6965313; doi:10.3389/fpls.2019.01697)
Supplement: Supplementary file 2 [file DataSheet_1.docx]

**Table S1**: Converted qPCR data (#nematodes/100g soil) on different nematode taxa for the three different soil management types.

| **Nematode taxon** | **ConMin** | **ConSlu** | **Org** |
| --- | --- | --- | --- |
| Dorylaimida | 11.55 | 28.52 | 8.08 |
| Heterorhabditidae | 0.01 | 0.01 | 0.37 |
| *Tylenchorhynchus* | 28.18 | 11.61 | 1.77 |
| *Coslenchus* | 0.00 | 0.00 | 1.09 |
| *Pratylenchus penetrans* | 0.00 | 0.00 | 1.81 |
| *Meloidogyne incognita* | 0.00 | 0.00 | 0.00 |
| *Meloidogyne chitwoodi* | 57.70 | 64.76 | 1.96 |
| *Meloidogyne fallax* | 0.47 | 0.00 | 0.00 |
| *Meloidogyne minor* | 0.00 | 0.00 | 0.00 |
| *Meloidogyne naasi* | 0.15 | 3.12 | 0.55 |
| *Meloidogyne hapla* | 0.00 | 0.00 | 0.00 |
| Total nematode abundance | 1551 | 1622 | 1440 |

**Table S2**: Summary of the PERMANOVA based on Bray-Curtis and UniFrac (Weighed and Unweighted) distances for DNA and RNA seperately. Sample type (Bulk/Rhizosphere), Treatment (ConSlu, ConMin, Org) and Time point (Vegetative/Generative) as factors. Differences are considered significant if P <0.01.

|  | DNA | | | | | | RNA | | | | | |
| --- | --- | --- | --- | --- | --- | --- | --- | --- | --- | --- | --- | --- |
|  | Bray Curtis | | Unifrac (U) | | Unifrac (W) | | Bray Curtis | | Unifrac (U) | | Unifrac (W) | |
| **Source** | **R^2^** | **P** | **R^2^** | **P** | **R^2^** | **P** | **R^2^** | **P** | **R^2^** | **P** | **R^2^** | **P** |
| **Bacteria** |  |  |  |  |  |  |  |  |  |  |  |  |
| Treatment | 0.101 | 0.001 | 0.097 | 0.001 | 0.061 | 0.001 | 0.103 | 0.001 | 0.125 | 0.001 | 0.055 | 0.001 |
| Sample Type | 0.378 | 0.001 | 0.185 | 0.001 | 0.672 | 0.001 | 0.428 | 0.001 | 0.215 | 0.001 | 0.740 | 0.001 |
| Time Point | 0.048 | 0.001 | 0.032 | 0.001 | 0.029 | 0.003 | 0.063 | 0.001 | 0.037 | 0.001 | 0.030 | 0.002 |
| Residuals | 0.472 |  | 0.686 |  | 0.238 |  | 0.406 |  | 0.622 |  | 0.174 |  |
| **Fungi** |  |  |  |  |  |  |  |  |  |  |  |  |
| Treatment | 0.154 | 0.001 | 0.116 | 0.001 | 0.110 | 0.001 | 0.165 | 0.001 | 0.154 | 0.001 | 0.137 | 0.001 |
| Sample Type | 0.218 | 0.001 | 0.074 | 0.001 | 0.295 | 0.001 | 0.208 | 0.001 | 0.117 | 0.001 | 0.236 | 0.001 |
| Time Point | 0.047 | 0.001 | 0.039 | 0.001 | 0.045 | 0.001 | 0.063 | 0.001 | 0.048 | 0.001 | 0.070 | 0.001 |
| Residuals | 0.581 |  | 0.771 |  | 0.550 |  | 0.565 |  | 0.682 |  | 0.557 |  |
| **Protozoa** |  |  |  |  |  |  |  |  |  |  |  |  |
| Treatment | 0.139 | 0.001 | 0.205 | 0.001 | 0.258 | 0.001 | 0.132 | 0.001 | 0.156 | 0.001 | 0.149 | 0.001 |
| Sample Type | 0.241 | 0.001 | 0.182 | 0.001 | 0.310 | 0.001 | 0.159 | 0.001 | 0.151 | 0.001 | 0.130 | 0.001 |
| Time Point | 0.136 | 0.001 | 0.086 | 0.002 | 0.149 | 0.001 | 0.120 | 0.001 | 0.060 | 0.001 | 0.145 | 0.001 |
| Residuals | 0.484 |  | 0.527 |  | 0.282 |  | 0.589 |  | 0.633 |  | 0.577 |  |
| **Metazoa** |  |  |  |  |  |  |  |  |  |  |  |  |
| Treatment | 0.138 | 0.001 | 0.125 | 0.001 | 0.065 | 0.007 | 0.101 | 0.001 | 0.141 | 0.001 | 0.057 | 0.004 |
| Sample Type | 0.168 | 0.001 | 0.091 | 0.001 | 0.225 | 0.001 | 0.200 | 0.001 | 0.172 | 0.001 | 0.302 | 0.001 |
| Time Point | 0.064 | 0.001 | 0.030 | 0.006 | 0.076 | 0.001 | 0.067 | 0.001 | 0.048 | 0.002 | 0.059 | 0.001 |
| Residuals | 0.630 |  | 0.754 |  | 0.634 |  | 0.632 |  | 0.639 |  | 0.582 |  |
